# Prevalence of antimicrobial resistance in urine, blood, and wound pathogens among rural patients in Karnataka, India

**DOI:** 10.1017/ash.2023.162

**Published:** 2023-05-15

**Authors:** Markos Mardourian, Hannah Lyons, Jackson Rhodes Brunner, Matthew K. Edwards, Archibald Lennox, Sumana Mahadevaiah, Sunitha Chandrashekhar, Suvvada Prudhvi Raj, Anjali Pradhan, Gautam Kalyatanda

**Affiliations:** 1 University of Florida College of Medicine, Gainesville, Florida, United States; 2 Case Western Reserve University School of Medicine, Cleveland, Ohio, United States; 3 Malcolm Randall Department of Veterans’ Affairs Medical Center, Gainesville, Florida, United States; 4 Department of Microbiology, JSS Medical College, Mysore, Karnataka, India; 5 Department of Pharmacy, Rhodes University, Grahamstown, South Africa; 6 JSS College of Pharmacy, JSS Academy of Higher Education and Research, Mysore, Karnataka, India; 7 Division of Infectious Disease and Global Medicine, University of Florida, Gainesville, Florida, United States

## Abstract

**Background and objective:**

Antimicrobial resistance (AMR) is increasing in tertiary-care hospitals across India, which consumes more antibiotics than any other country. Microorganisms with novel resistance mechanisms, initially isolated in India, are now recognized worldwide. Until now, most efforts to stem AMR in India have focused on the inpatient setting. Ministry of Health data now suggest that rural areas are playing a more significant role in the pathogenesis of AMR than was previously appreciated. Thus, we conducted this pilot study to ascertain whether AMR is common in pathogens causing infections acquired in the wider rural community.

**Methods:**

We performed a retrospective prevalence survey of 100 urine, 102 wound, and 102 blood cultures obtained from patients who were admitted to a tertiary-care facility in Karnataka, India, with infections acquired in the community. The study population included patients >18 years of age who (1) were referred to the hospital by primary care doctors, (2) had a positive blood, urine, or wound culture, and (3) were not previously hospitalized. Bacterial identification and antimicrobial susceptibility testing (AST) were carried out on all isolates.

**Results:**

*Enterobacteriaceae* were the most common pathogens isolated from urine and blood cultures. Significant resistance to quinolones, aminoglycosides, carbapenems, and cephalosporins was noted among pathogens isolated from all cultures. Specifically, high resistance rates (>45%) to quinolones, penicillin, and cephalosporins were evident among all 3 types of culture. Among blood and urinary pathogens, there were high resistance rates (>25%) to both aminoglycosides and carbapenems.

**Conclusion:**

Efforts to stem AMR rates in India need to focus on rural populations. Such efforts will need to characterize antimicrobial overprescribing practices, healthcare-seeking behaviors, and antimicrobial use in agriculture in rural settings.

Antimicrobial resistance (AMR) poses a threat to the treatment of infectious conditions and leads to an increase in morbidity, mortality, disease transmission, and healthcare costs. In 2019, bacterial AMR was associated with ∼4.95 million deaths globally, with the highest rates of mortality reported in sub-Saharan Africa and South Asia.^1^ Disparities in AMR morbidity and mortality are multifactorial. High rates of AMR often require the use of more expensive, “last resort” antibiotics, which presents a significant challenge in low- and middle-income countries (LMICs).^2,3^ Despite significant progress with AMR surveillance in recent years, LMICs in South and Southeast Asia continue to struggle with the establishment and expansion of regulatory programs due to technical, financial, and logistical constraints in healthcare infrastructure.^4^

India has one of the highest burdens of bacterial infection in the world, and its population consumed more antibiotics than any other country from 2000 to 2010.^3,5,6^ Various highly resistant microorganisms with novel resistance mechanisms, such as the New Delhi metallo-β-lactamase enzymes in *Enterobacteriaceae*, were initially isolated in India and are now recognized worldwide.^5^ Due to the gravity of this situation, India’s Ministry of Health and Family Welfare declared AMR a national health priority and, in conjunction with the World Health Organization (WHO), developed the National Action Plan on Antimicrobial Resistance (NAP-AMR) for 2017–2021.^7^ Six strategic priorities were identified in this action plan, including strengthening of knowledge and evidence through AMR surveillance, reduction in the incidence of infection through effective prevention and control policies, and the optimization of antimicrobial use in healthcare, animal domestication, and food production.^7^

Significant AMR to firstline therapeutic agents (ie, β-lactams, cotrimoxazole, and fluoroquinolones) among gram-negative microorganisms in India has led to their replacement by broad-spectrum agents (ie, carbapenems and aminoglycosides). AMR has now been documented in urinary pathogens in Indian hospitals servicing both urban and rural populations, and decreasing effectiveness of “last resort” antimicrobial agents like carbapenems is an alarming phenomenon.^3,8–11^ Susceptibility testing in commensal microorganisms indicate that AMR in India is widespread in rural communities outside the tertiary hospital setting.^12–14^ Specifically, Singh et al^3^ found multidrug resistance in >25% of the commensal *Escherichia coli* isolated from patients in rural villages of northeast India. Furthermore, researchers identified variation in resistance patterns between villages within the same state, suggesting a greater degree of diversity in resistance mechanisms than initially anticipated.^3^ Although these studies of commensal organisms provide valuable approximations, they likely underestimate the resistance rates among pathogenic microorganisms isolated from clinical specimens. Patients from rural areas admitted to tertiary-care centers in India often already harbor pathogens that are resistant to available antimicrobial agents.

Although many reports have documented AMR among clinical isolates in the tertiary-care setting in India, there is a paucity of similar data from the primary healthcare centers (PHCs) and rural communities in India.^12^ Because these data have suggested that primary healthcare settings in rural areas might be playing a more significant role in the pathogenesis of AMR than previously appreciated, we conducted a pilot survey as part of the first phase of a larger study (1) to characterize the resistance profiles of infections caused by sentinel, antimicrobial-resistant pathogens causing infections in the rural setting and (2) to estimate the extent of the AMR among rural patients referred from a primary to a tertiary-care center for advanced care.

## Methods

We carried out a retrospective prevalence survey of urine, wound, and blood cultures obtained from patients admitted to the the Jagadguru Shivarthreeshwara Hospital in Karnataka, South India, during January and February 2021. The hospital is a tertiary-care facility with 1,800 general medical beds and ∼260 critical care beds. It caters to the healthcare needs of the rural population in the Mysore district and the population of Mysore city.

The study population included patients aged >18 years who were referred to the hospital from surrounding rural village with infections acquired in the urinary tract, wounds, or bloodstream. We focused on isolates from patients with urine, wound, and blood infections because of the relatively high predictive value for positive cultures from these anatomic sites.

Patients were enrolled in the study after informed consent if they (1) lived in a rural area, (2) acquired their infections in the community before referral to the tertiary hospital, and (2) had not been hospitalized previously.

The case definitions for obtaining cultures and selecting study participants were as follows:

1. Urinary tract infection: any patient over the age of 18 who presented with an oral temperature ≥38°C, burning on micturition, dysuria, urine analysis with pyuria and a midstream urine specimen that yielded growth on culture.
2. Bacteremia: any patient over the age of 18 who presented with signs and symptoms suggestive of systemic infection and had a positive blood culture.
3. Wound infection: any patient over the age of 18 with cultures obtained from skin abscesses or from purulent discharge from the wound.

All cases were ascertained by chart review. We obtained approval from the Institutional Review Board at the University of Florida and the Local Ethics Committee at the study hospital.

Bacterial identification and antimicrobial susceptibility testing (AST) were carried out on the VITEK 2 fully automated system (BioMérieux, Marcy-l’Étoile, France) that uses AST cards based on the broth microdilution minimum inhibitory concentration platform according to the benchmarks established by the Clinical and Laboratory Standards Institute (CLSI) in the United States. The Jagadguru Shivarthreeshwara Hospital Microbiology Laboratory, where our study isolates were cultured, provides microbiology services for the hospital but is also a national reference laboratory that has links with the World Health Organization (WHO), the CDC, and clinical laboratories across countries in the British Commonwealth.

## Results

During the study period, 100 urine cultures, 102 blood cultures, and 102 wound cultures yielded growth.

### Urine

*Escherichia coli* was the most frequently isolated pathogen (47%), followed by *Enterococcus* spp (13%) and *Candida* spp (15%) (Table 1). A resistance rates >60% were detected for cephalosporins, quinolones, and penicillin (Table 2). Resistance rates >25% among urinary pathogens were also documented for aminoglycosides. A relatively high rate of resistance to carbapenems (>25%) was also documented. Colistin was sensitive in 95% of strains of *Acinetobacter baumannii*, *Enterobacter* spp, *Escherichia coli*, and *Klebsiella pneumoniae*.

**Table 1.** Positive Cultures by Cultures by Organism and Culture Site

Organism	Culture Site		
	Wounds, No. (%)	Bloodstream, No. (%)	Urinary Tract, No. (%)
*Acinetobacter* spp	0	10 (10.0)	3 (3.0)
*Candida* spp	0	2 (2.0)	15 (15.0)
*Citrobacter* spp	5 (4.3)	0	1 (1.0)
Coagulase-negative *staphylococcus*	16 (13.0)	22 (22.0)	4 (4.0)
*Enterobacter* spp	3 (2.6)	3 (3.0)	2 (2.0)
*Enterococcus* spp	6 (5.2)	10 (10.0)	13 (13.0)
*Escherichia coli*	15 (13.0)	15 (15.0)	47 (47.0)
*Klebsiella pneumoniae*	16 (14.0)	19 (19.0)	9 (9.0)
*Pseudomonas aeruginosa*	15 (13.0)	3 (3.0)	4 (4.0)
*Salmonella* ser. *typhi*	0	5 (5.0)	0
*Serratia* spp	0	4 (4.0)	
*Staphylococcus aureus*	26 (22.4)	3 (3.0)	0
Streptococcus spp	5 (4.3)	3 (3.0)	0
Other Enterobacteriaceae^a^	8 (6.9)	2 (2.0)	3 (3.0)
Other gram-negative nonfermenters^b^	1 (0.9)	1 (1.0)	0
Total	116	102	101

a *Morganella morganii*, *Pantoea agglomerans*, *Proteus* spp, and *Providencia* spp

b *Achromobacter denitrificans* and *Burkholderia cepacia.*

**Table 2.** Overall Resistance Profiles of Wound, Blood, and Urinary Pathogens by Antimicrobial Class

Drug Class	Wound Pathogens			Blood Pathogens			Urinary Pathogens		
	% Resistant	% Susceptible	No. of Organisms	% Resistant	% Susceptible	No. of Organisms	% Resistant	% Susceptible	No. of Organisms
Aminoglycosides	18	79	160	48	48	149	31	66	148
Azoles	0	100	1	0	1	4	4	96	26
Carbapenems	13	85	175	41	55	164	29	69	185
Cephalosporins	45	50	272	68	26	280	61	35	322
Echinocandins	0	0	0	0	100	4	0	100	26
Macrolides	16	84	80	68	28	40	88	13	8
Penicillins	47	45	213	72	26	202	61	35	208
Quinolones	65	33	209	65	30	182	85	14	155
Tetracyclines	19	79	52	19	80	118	25	68	60
Polymyxins	12	0	51	27	72	318	5	0	38
Others	23	76	358	10	2	52	25	68	214

Note: The remaining percentages for each drug class reflect intermediate susceptibility.

### Blood

The most frequently isolated microorganisms were coagulase-negative *Staphylococcus* spp (22%), *K. pneumoniae* (19%), and *E. coli (*15%) (Table 1). Resistance rates were >65% for cephalosporins, quinolones, and penicillin (Table 2). High resistance rates were also noted for aminoglycosides (48%) and carbapenems (41%). Most (88.5%) of the isolates also displayed intermediate sensitivity to colistin.

### Wounds

*Staphylococcus aureus* was the most frequently isolated organism (22.4%), followed by *Klebsiella* pneumoniae (14%), whereas coagulase-negative *Staphylococcus, E. coli,* and *Pseudomonas aeruginosa* each comprised 13% of the wound isolates (Table 1). Almost one-fifth of all organisms isolated were multidrug resistant (17.1%), and the highest rates of resistance were attributed to quinolones (65%), penicillins (47%), and cephalosporins (45%) (Table 2). The resistance rates for aminoglycosides and carbapenems were 18% and 13%, respectively.

The antimicrobial resistance profiles of the most common microorganisms isolated from wounds, bloodstream, and urine in the study population are summarized in Tables 3a and 3b.

**Table 3a.** Percentage Resistance to Available Antimicrobial Agents Among Common Gram-Negative Microorganisms Isolated From Wounds, Blood, and Urine Obtained From Patients in Rural Areas

Antimicrobial Agent	*Acinetobacter* spp	*Escherichia coli*	*Enterobacter* spp	*Klebsiella* spp	*Pseudomonas* spp	*Salmonella* spp
Aminoglycoside	76	35	50	58	14	100
Amoxicillin	100	49	100	66	…	0
Ampicillin	100	90	25	100	…	0
Carbapenem	92	18	25	56	0	0
Cefepime	91	38	38	66	10	0
Ceftazidime	90	…	…	…	13	…
Ceftriaxone	100	75	63	85	…	…
Nitrofurantoin.	100	2	50	79	…	…
Piperacillin	91	35	50	84	4	0
Quinolone	80	92	50	84	32	100
TMP-SMX	69	51	57	64	…	…
Polymyxin B	7	0	0	0	0	0

Note. TMP-SMX, trimethoprim-sulfamethoxazole.

**Table 3b.** Percentage Resistance to Available Antimicrobial Agents Among Common Gram-Positive Microorganisms Isolated From Wounds, Blood, and urine Obtained From Patients in Rural Areas

Antimicrobial Agent	*Enterococcus* spp	*Staphylococcus aureus*	*Streptococcus* spp
Aminoglycoside	56	40	*…*
Amoxicillin	100	100	0
Ampicillin	62	*…*	0
Ceftriaxone	*…*	*…*	50
Glycopeptide	12	0	0
Macrolide	60	46	40
Nitrofurantoin	42	*…*	*…*
Oxacillin	*…*	56	*…*
Penicillin	73	*…*	57
Quinolone	83	85	
TMP-SMX	80	30	100
Tetracycline	73	6	0

Note. TMP-SMX, trimethoprim-sulfamethoxazole.

## Discussion

High rates of antimicrobial resistance portend poor patient outcomes. Our study confirmed a high prevalence of resistance among clinical isolates at a tertiary-care hospital serving a large catchment of rural communities. Similar to surveillance data on healthcare-associated infections published by the US Centers for Disease Control and Prevention (CDC),^15^ *E. coli, Enterococcus* spp, and *Candida* spp were the 3 most common causes of symptomatic UTIs in this population. However, the CDC data were drawn from critical care patients with indwelling urinary catheters in US tertiary-care hospitals, and the specimens in the current study were obtained from symptomatic patients from rural areas. Previous studies have shown *E. coli* to be the most common pathogen in both rural and urban populations. We found high rates of resistance of urinary pathogens to aminoglycosides, as has been described in previous studies.^16^

Other studies have shown that *E. coli* isolates from the community tend to have high overall resistance to ampicillin (75%), nalidixic acid (73%), and trimethoprim-sulfamethoxazole (59%).^17^ In a 2019 study performed in rural South India, ∼3% of Enterobacteriaceae were resistant to nonertapenem carbapenems, whereas resistance to ertapenem was as high as 4 times that of other carbapenems.^18^ In India, from 2008 to 2018, *E. coli* resistance to third-generation cephalosporins increased from 71% to 88% and fluoroquinolone resistance increased from 83% to 89%, whereas carbapenem resistance increased from 9% to 41%.^19,20^ Similar to the resistance patterns cited from other studies, we detected a resistance rate >60% for cephalosporins and quinolones. Additionally, a relatively high rate of resistance (>25%) to carbapenems has also been documented.

In a prior study comparing AMR in rural and urban areas in South India, most isolates from patients in urban centers are gram-negative pathogens; *E. coli* and *Klebsiella* spp are the most common, and *S. aureus* comprises only 4% of specimens. By comparison, *E. coli* and *S. aureus* were the most common pathogens in rural areas (30%). More than 25% of all *K. pneumoniae* and *Pseudomonas* spp isolates showed resistance to imipenem, and a similar proportion of all *E. coli, Klebsiella* spp and *Pseudomonas* spp isolates were resistant to fourth-generation cephalosporins.^16^

In this study, *S. aureus* (including MRSA) was the most common pathogen causing wound infections, with ∼20% of all bacteria being multidrug resistant (ie, resistant to ≥2 antimicrobial agents). Coagulase-negative staphylococci constituted ∼36% of all wound and blood cultures, likely representing colonization rather than true infection. Gram-negative microorganisms, including *E. coli* and *Pseudomonas* spp, were also leading causes of wound infections as well as bloodstream infections, in which nearly 40% of isolates were resistant to >1 carbapenem. The large number of coagulase-negative staphylococci isolated from blood cultures were almost certainly a result of blood-culture contamination because they represent the most common blood-culture contaminants.^21^ Studies have shown that the mortality rate is higher in patients who are infected by *A. baumannii*. In a previous study of ICU patients with bloodstream infections, the mortality rate was twice as high among those with gram-negative MDR infections by *E. coli*, *K. pneumonia*, and *Acinetobacter* compared to those infected with non–drug-resistant pathogens.^22^ In the current study, *Acinetobacter* spp constituted ∼10% of all bloodstream infections.

In the tertiary-care setting, risk factors associated with AMR include frequent use of invasive devices, prescription of empiric antimicrobials, critically ill patient population, high patient census, and opportunities for cross transmission during close contact between ICU personnel and their inpatient population. These AMR risk factors have long been characterized in myriad published studies of tertiary-care centers in economically developed countries.^23–28^ A combination of these risk factors with a failure to identify patients infected or colonized with AMR pathogens is the usual reason for increased resistance in the healthcare setting.

In rural areas, antimicrobial overprescribing represents one of the most significant contributors to AMR because patients in these areas are less likely to have in situ urinary catheters, intravascular catheters, or other invasive medical devices. Because patients in rural settings in India usually present to the primary care setting in the first instance; thus, it is highly probable that rural populations already harbor resistant strains of *Staphylococcus* spp, *Enterobacteriaceae*, or nonfermenters, such as *Pseudomonas* spp and *Acinetobacter* spp. This reality then leads to the following question: Is the emergence of AMR among strains of healthcare-associated pathogens seen in tertiary-care centers in India a consequence of prescribing practices in rural areas? Unfortunately, published data pertaining to this hypothesis are scarce. It remains unknown whether unique risk factors peculiar to the rural setting exist, such as use of antimicrobials as growth promoters in farming and other agricultural endeavors. Hence, there is a need for a paradigm change from focusing on tertiary-care hospitals to focusing on primary care settings.

This study had several limitations. Its design was descriptive; there was no collection of data regarding clinical or epidemiologic risks factors or patient outcomes. By intention, however, we pilot-tested a project to ascertain the presence of AMR in this tertiary-care setting. In the next phase of our project, we plan to perform a prospective, epidemiologic study that characterizes AMR in rural populations to understand attributable risk factors and clinical surrogate markers.

Most of rural India’s population visits government primary healthcare centers for their medical needs. Guidelines to treat common infections are based on recommendations that do not consider the local antibiograms. Rural primary healthcare centers lack the infrastructure to perform cultures and sensitivities. Furthermore, laboratory technicians often lack the expertise to perform these tests.

The NAP-AMR strategy prioritizes the strengthening of microbiology laboratories and establishing standards for AMR surveillance in humans, animals, food, and the environment.^7^ Although the Indian Council of Medical Research has initiated a network to provide surveillance data at the national level, very little is known about AMR in rural communities and PHCs. Future studies should examine the contribution of prescription practices, healthcare-seeking behaviors, and antimicrobial use in agriculture to the locally high rates of antimicrobial resistance.

Antimicrobial resistance affects the socioeconomic growth, food security, environmental health, and long-term financial viability of communities.^29,30^ Unless AMR is tackled at the level of the primary healthcare center, its severe social and economic effects will continue to push India’s citizens into poverty. This pilot study was the first phase of a planned long-term endeavor to characterize resistance in healthcare centers throughout the rural and urban communities of India.

In conclusion, the current study has demonstrated a high prevalence of resistance among clinical isolates obtained from urine, blood, and wound cultures at a tertiary-care hospital serving a large catchment of rural communities. Specifically, high resistance rates (>45%) to quinolones, penicillin, and cephalosporins were evident among all three types of cultures. The gradual replacement of what used to be considered firstline therapeutic agents by broad-spectrum agents, such as carbapenems and aminoglycosides, represents an ongoing challenge in India. This issue has led to poor antimicrobial stewardship, especially in primary healthcare settings where resources are scarce and antimicrobial overprescribing is less subject to established antimicrobial stewardship programs. Our findings revealed another alarming problem regarding these broad-spectrum antimicrobials that were once viewed as last resort agents. Among blood and urinary pathogens, resistance rates to both aminoglycosides and carbapenems were high (>25%). Thus, despite being considered last-resort agents, these agents might become increasingly ineffective in the treatment of resistant infections under current prescription practices. AMR is not a phenomenon restricted to tertiary-care centers, and it represents a significant challenge in rural settings. There is a relative lack of literature focusing on AMR in rural healthcare settings in India. This study highlights the need to conduct future studies investigating the contribution of prescription practices, healthcare-seeking behaviors, and antimicrobial use in agriculture to the alarming rates of AMR in rural settings.
